# Colorimetric sensing of imidacloprid in cucumber fruits using a graphene quantum dot/Au (III) chemosensor

**DOI:** 10.1038/s41598-020-71349-4

**Published:** 2020-08-31

**Authors:** Saeedeh Babazadeh, Parviz Ahmadi Moghaddam, Sajjad Keshipour, Kaveh Mollazade

**Affiliations:** 1 Department of Mechanical Engineering of Biosystems, Faculty of Agriculture, Urmia University, 11 km Sero Road, Urmia, Iran; 2 Department of Nanochemistry, Nanotechnology Research Center, Urmia University, Shahid Beheshti St., Urmia, Iran; 3 Department of Biosystems Engineering, Faculty of Agriculture, University of Kurdistan, Sanandaj, Iran

**Keywords:** Food nanotechnology, Sensors

## Abstract

The current research presents a very simple method for the colorimetric detection of imidacloprid using a graphene quantum dot/Au (III) chemosensor. The results demonstrated that there is an interaction between Au^3+^ ions and the imidazole group of the pesticide toward reduction of Au^3+^ to Au^0^ in the presence of graphene quantum dots. This phenomenon changes the color of gold nanoparticles from yellow to grey or red, and causes a shift in the peak of localized surface plasmon resonance (LSPR) as gold nanoparticles are formed or aggregated based on the concentration of imidacloprid. Imidacloprid was determined by the developed sensor in a linear area of 0.01–1 ppm with a detection limit of 0.007 ppm. Therefore, a simple, quick, and sustainable sensor has been developed for the determination of the investigated analyte. Moreover, the sensor was applied to determine imidacloprid in the real cucumber samples fairly successful.

## Introduction

Insecticides have a vital role in protecting plants from insects and pests. Neonicotinoid insecticides are increasingly being replaced by organophosphorus and carbamate pesticides to control sucking pests^1^. Within the neonicotinoid pesticide group, imidacloprid (*N*-{1-[(6-chloro-3-pyridyl)methyl]-4,5-dihydroimidazol-2-yl}nitramide) is the second pesticide in terms of popularity in the world^2^. It has adverse effects on human health. In recent years, the excessive use of pesticides has caused an increase in their residues in agricultural products, especially in greenhouse products such as cucumber and tomato. For this reason, acceptable daily intakes (ADIs) and maximum residue levels (MRLs) were established by international organizations as the criteria to prevent the adverse health effects of pesticide residues^3^. The MRL and ADI for imidacloprid in cucumbers are set to 1 mg/kg and 0.06 mg/kg of body weight every day, respectively^4^. Currently, a variety of methods, such as gas chromatography (GC), liquid chromatography–mass spectrometry (LC–MS), high-performance liquid chromatography (HPLC), the enzyme-linked immunosorbent assay, and electrochemical methods have been employed in the detection of imidacloprid^1^. Although these methods are highly accurate, some disadvantages, such as high cost, onerous time requirements, and unreliability for real-time detection make them suitable for rapid measurements. So it is necessary to develop new and reliable methods, such as colorimetric methods, for rapid detection of imidacloprid.

Nanotechnology is an interdisciplinary area of research that has grown globally in the last few decades^5^. Among nanostructures, graphene quantum dots (GQDs) and gold nanoparticles (AuNPs) have attracted particular attention^6^. GQDs are the sheets of graphene with the size of less than 100 nm that have same properties to graphene^7^. Recently, GQDs were used as reducing agents for synthesizing metallic nanoparticles in sensor applications. Amjadi et al*.*^8^ applied GQDs as the reducing agent for the construction of a GQD/Ag nanocomposite.

Gold nanoparticles are used in producing ocular sensors through their high stability and ease of use^9^. AuNPs are popular to show various colors with changes in their size^10^. AuNP-based colorimetric sensors is so appropriate for on-site and fast detection of imidacloprid in places where resources are limited due to its highly sensitive detection and its advantages of simplicity, short time requirement, and its minimal use of equipment^9^. AuNPs were used in the area of pesticide detection, especially in agricultural crops. The sensitivity of these nanoparticles can be improved by modifying their surfaces for detecting different pesticides. Different organophosphorous and organochlorine insecticides have been determined using AuNP-based sensors^10^. The colorimetric recognition of imidacloprid using AuNPs is a rapid, ocular, easy to use, and on-site method^1^. Zhang et al*.*^11^ used AuNPs as a colorimetric sensor for detecting imidacloprid in surface-water samples.

*N*-Heterocyclic carbenes (NHCs) are ligands that form strong links with the high-oxidation-state metals such as gold. These covalent connections of NHCs with gold lead to obtain AuNPs with a high colloidal stability^12^. Some *N*-heterocyclic compounds, such as imidazole and tetrazole, which are called ligands, readily donate electrons in the formation of organometallic complexes. These complexes have shown particular use in the construction and stabilization of AuNPs^13^. Moraes et al*.*^14^ synthesized very stable AuNPs through *N*-heterocyclic-2-thiones. Moreover, Lu et al*.*^15^ reported that the imidazole is an electron-donor; thus, it can conduct electrons to gold. Nazirov et al*.*^16^ synthesized AuNPs ($d=2.3$ nm) which are soluble in water. The reduction procedure of Au (III) was done using *N*-(4-imidazolyl) methyl-chitosan that is a biocompatible reducing agent. It is important to mention that AuNPs are solely formed by adding a reductant in solutions that include an imidazole-containing polymer and imidazolium-based ionic liquids.

The current research provides a very simple way to determine imidacloprid calorimetrically by applying a graphene quantum dot/Au (III) chemosensor (GQD/Au^3+^). The interaction between the GQD/Au^3+^ composite and the imidazole group of the pesticide form AuNPs. The sensor has been applied to detect imidacloprid in stock and real specimen. The innovation aspect of the current research is being first study that can recognize the presence of imidacloprid with the naked eye using a GQD/Au^3+^ sensor without the addition of any toxic reducing agent. In this regard, the aim of the present research is to visually detect the residue of imidacloprid in standard samples and greenhouse-grown cucumbers using an on-site sensor based on a GQD/Au (III) colorimetric sensor.

## Results and discussion

### Characterization of GQDs

GQDs were characterized using UV–Vis spectrophotometry, FT-IR spectroscopy, and transmission electron microscopy (TEM). The absorption spectrum of the GQD solutions in water indicated two distinct absorption peaks (Supplementary Fig. S1): a main peak at the wavelength of 400 nm and a shoulder at 340 nm; these are the peaks of GQDs which are related to the π–π^*^ and the n–π^*^ electron transitions of C=C and C=O bonds, respectively^8^.

Supplementary Fig. S2 shows the characteristic peaks of the synthesized GQDs’ FT-IR spectrum. The corresponding wavenumber at 3,345 cm^−1^ belongs to the vibration modes of hydroxyl (–OH) groups^17^. The stretch vibration of aliphatic C–H groups was observed at around 2,947 cm^−118^. In addition, the wavenumbers at 1,703, 1,649, and 1,015 cm^−1^ are regarded to the stretch vibrations in C–O^19^, C–C^17^, and C–O^18^ groups, respectively. These peaks show that the GQDs were surrounded by carboxyl and hydroxyl functional groups^20^. Thus, the hydroxyl groups of GQDs can be used as reducing agents for the chemical synthesis of nanostructures^8^.

The TEM micrograph of spherical GQDs indicates a homogeneous formation of the nanostructures. It also shows that the particle-size contribution of the constructed GQDs was between 8 and 14 nm (Supplementary Fig. S3).

### Formation of AuNPs by the pesticide’s imidazole group

As mentioned above, AuNPs can be formed in the presence of the insecticide’s imidazole group. To confirm this, the absorption spectra of the sensor in the absence and presence of the pesticide were recorded. There is a small LSPR peak in the absence of pesticide after half an hour (Supplementary Fig. S4a); in contrast, an LSPR peak in the range of 532–567 nm can be seen in the complex of GQD/Au^3+^/imidacloprid according to the concentration of imidacloprid. This peak, which is characteristic of AuNPs, shows the formation of gold nanoparticles in the solution. The formation of AuNPs can also be determined from changes in color (Supplementary Fig. S4b), showing that GQDs act as a very weak reducing agent, but that the pesticide’s imidazole group accelerates the formation of AuNPs in the first minutes.

### Investigation of GQDs and Au (III) solutions in the detection of imidacloprid

For developing a colorimetric sensor to detect imidacloprid, the influence of synthesized GQDs (0.1 g/ml) and prepared Au^3+^ (0.001 M) solutions was studied solely in the detection of imidacloprid. The results of GQDs application in the presence of imidacloprid are shown in Supplementary Fig. S5a. Even if there are not any changes in the absorbance of GQDs at different concentrations of imidacloprid (0–20 µM), there is an increase at the wavelength of 271 nm which is the characteristic peak of imidacloprid (Supplementary Fig. S5b).

Moreover, the absorbance of Au (III) solution in the presence of three concentrations of pesticide (0.1, 1, and 10 ppm) is demonstrated in Supplementary Fig. S6. The reduction of Au (III) was reported with various heterocycles^14,16^ and imidacloprid also, as a heterocyclic compound, can do that. GQD as a compound with a lot of π electrons just accelerates the electron convey between imidacloprid and Au (III).

### Colorimetric detection of imidacloprid

**Figure 1:**
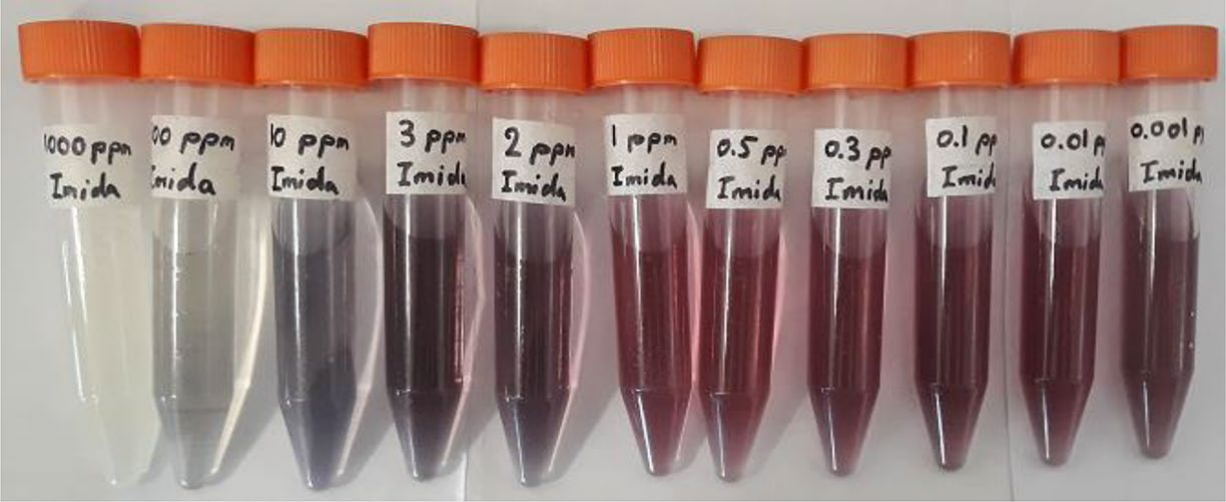
The images of AuNPs with different concentrations of imidacloprid between 0.001 and 1,000 ppm.

The detection of imidacloprid was performed using a GQD/Au^3+^ sensor on two categories of the insecticide samples: standard solutions of the insecticide and real samples of cucumbers harvested on various days after having been sprayed with imidacloprid. The GQD/Au^3+^ composite acted as a colorimetric sensor, which showing distinct color changes in the presence of imidacloprid residues as a result of Au^3+^ reduction to gold nanoparticles promoted with GQD/imidacloprid. The color differences are attributed to the formation of gold particles with different size distributions, which is in turn related to the concentration of imidacloprid. For concentrated solutions, the reduction process of Au^3+^ was performed in an acidic medium, which triggered the aggregation of particles at high concentrations^21^; for diluted solutions, particles were formed in the low rate and far from together, leading to fine particles. The color change using GQD/Au^3+^ was investigated on various concentrations of imidacloprid between 0.001 and 1,000 ppm. While the GQD/Au^3+^ changed color after 20 min, it occurred after only 10 min for GQD/Au^3+^/imidacloprid; the mixture continued to darken even after 20 min compared to GQD/Au^3+^ (Supplementary Fig. S4b). As seen in Fig. 1, the formation of AuNPs in low concentrations of imidacloprid between 0.001 and 1 ppm caused the color to change from yellow to red. However, the color shifted to reddish-grey above concentrations of 1 ppm. At high concentrations of imidacloprid, particularly above 100 ppm and continuing to 1,000 ppm, an intense color change was not observed because at such high ratios of reducing agents to metallic ions, the formation of gold nanoparticles will be suppressed^8^.

**Figure 2:**
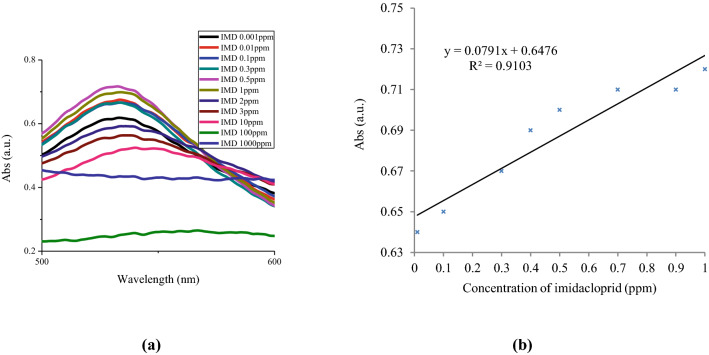
(**a**) UV–Vis spectra of AuNPs and (**b**) the corresponding calibration curves with different concentrations of imidacloprid (IMD) between 0.01 and 1 ppm.

The UV–Vis spectra were provided for the samples for which the characteristic peak attributed to gold nanoparticles was observed (Fig. 2a). The intensity of maximum absorbance increased as the pesticide concentration increased from 0.001 to 1 ppm, indicating incremental growth in the concentration of AuNPs^22^, while the absorbance intensity significantly decreased from 1 to 2 ppm, as indicated by the sudden color change of AuNPs from red to grey and increases in particle aggregation. At concentrations greater than 2 ppm, the absorbance intensity declined until it disappeared at 1,000 ppm. This confirms the suppression of AuNPs shown in Fig. 1. The developed sensor determined imidacloprid in the linear range of 0.01–1 ppm, with a detection limit (LOD) of 0.007 ppm and a limit of quantification (LOQ) of 0.025 ppm (Fig. 2b). Additionally, the calibration curve was linear, with a correlation coefficient (R^2^) of 0.9103.

An increase in the maximum absorbance wavelength (λ_max_) was associated with increases in the concentration of imidacloprid; this obviously indicates growth in the size of the gold nanoparticles (Supplementary Table S1). In direct relation to increases in the concentration of imidacloprid, the SPR peak of AuNPs shifted from 532 to 567 nm; consequently, the color changed from red to reddish-grey and then grey.

**Figure 3:**
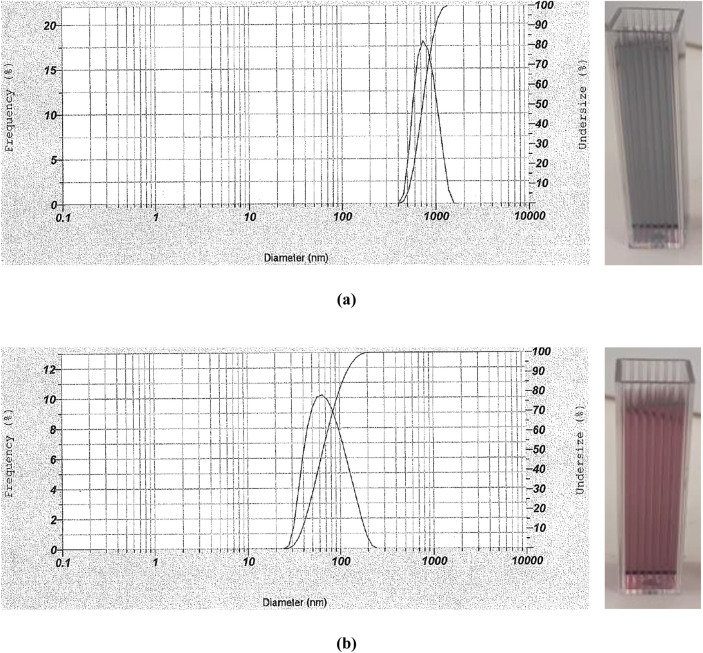
The images and DLS analyses of (**a**) imidacloprid 100 ppm and (**b**) imidacloprid 1 ppm after addition of the sensor.

To confirm the results, two dynamic light scattering (DLS) analyses were performed on the samples with two concentrations of pesticide—1 and 100 ppm—to determine gold particle sizes (Fig. 3). The results showed an average size of 730 and 65 nm for imidacloprid at 100 ppm and 1 ppm, respectively, confirming that the nanoparticle sizes increased at higher insecticide concentrations.

### Analysis of real samples

**Figure 4:**
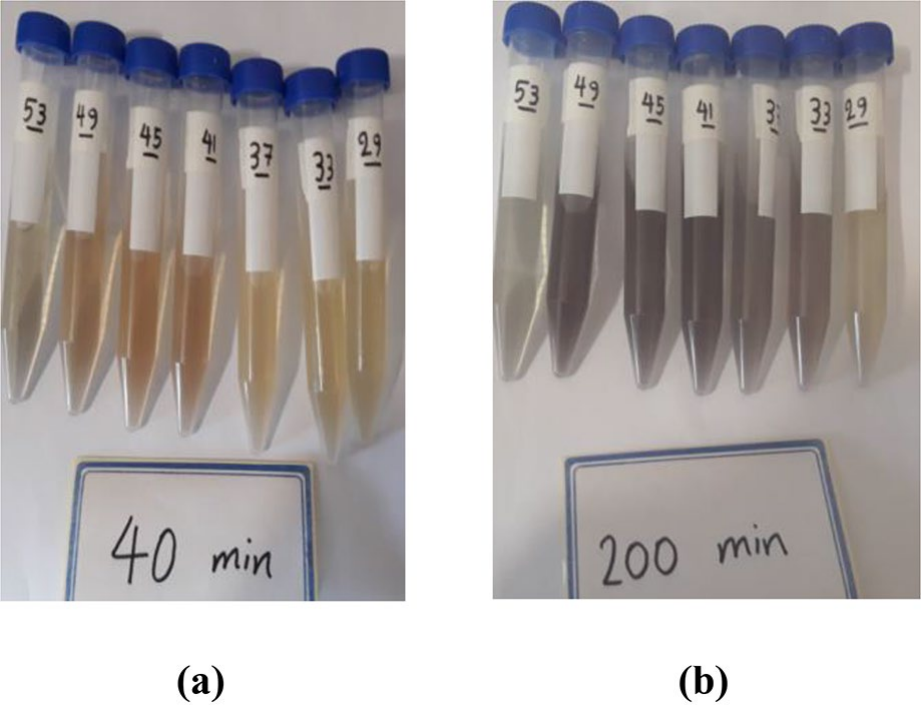
The images of real samples, right to left belongs to 1, 2, 4, 8, 15, 21, and 25 days after spraying, in the presence of GQD/Au^3+^ sensor after (**a**) 40 min and (**b**) 200 min.

Real samples were examined using the new sensor to evaluate the performance of GQD/Au^3+^ for natural samples. The extract of cucumbers harvested at 1, 2, 4, 8, 15, 21, and 25 days after being sprayed with imidacloprid were treated with GQD/Au^3+^ to evaluate pesticide levels using colorimetric detection. As seen in Fig. 4, samples during the pre-harvest interval (PHI) of imidacloprid, which is 21 days, showed relatively similar colors; the color of the sample taken after 25 days was markedly different. These results are completely in accordance with the pre-harvest interval of imidacloprid, and with the analogous data for the standard samples at the same PHI. The HPLC analyses confirmed these data that there is a less amount of pesticide near to zero after 21 days which makes the color different compared to the days before 21st day (Supplementary Table S2).

**Figure 5:**
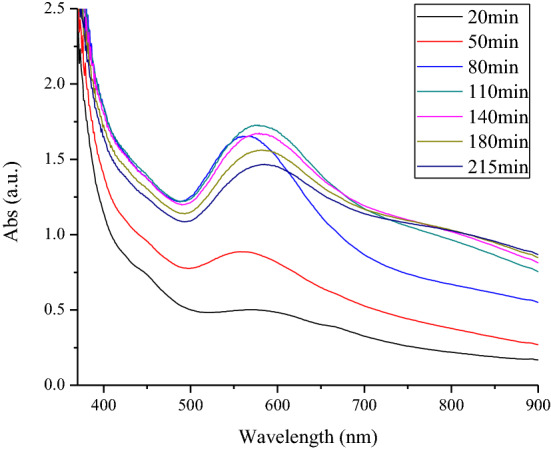
UV–Vis spectrum of sample number 45 during different contact times in the presence of GQD/Au^3+^ sensor.

UV–Vis spectra also confirmed the production of gold nanoparticles with the addition of the sensor to real samples after 10 min. The peak of gold nanoparticles appeared at about 530–570 nm according to the size of nanoparticles. In addition, a growth in λ_max_ was observed between the sensor and the real samples (Fig. 5), confirming that particle aggregation increased with longer reaction times^13^.

### The interference study of the novel sensor

**Figure 6:**
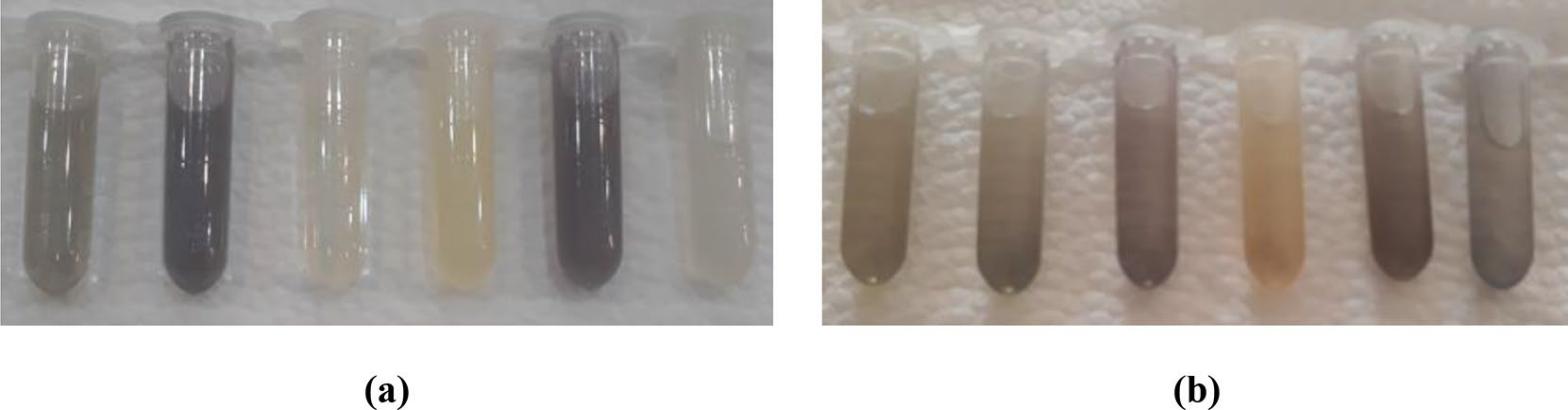
The interference study of colorimetric detection of imidacloprid by GQD/Au (III) sensor (From left to right: blank, only imidacloprid, imidacloprid & penconazole, imidacloprid & chlorpyrifos, imidacloprid & infinito, and imidacloprid & previcur energy) after (**a**) 1 h and (**b**) 1 day.

The interference studies of penconazole, infinito, previcur energy, and chlorpyrifos were performed on the synthesized sensor in the detection of imidacloprid. As shown in Fig. 6a, the sensor can recognize imidacloprid in the presence of infinito after 1 h. However, other studied pesticides such as chlorpyrifos, previcur energy, and penconazole were shown to interfere with the performance of the applied sensor; whereas, after passing 1 day from the reaction time (Fig. 6b), it was observed that the sensor does not have ability to recognize imidacloprid only in the presence of chlorpyrifos.

### The comparison of the GQD/Au^3+^ sensor’s detection of imidacloprid with some previous reports

Some methods, such as electrochemical and colorimetric methods, have been investigated for detecting imidacloprid using nanoparticles. Chen et al*.*^23^ studied the cyclic voltammetry (CV) and differential pulse voltammetry (DPV) methods for detecting imidacloprid using a β-cyclodextrin polymer functionalized reduced graphene oxide modified glassy carbon electrode in the linear ranges of 1–150 μM for CV and 0.05–150 μM for DPV. Majidi and Ghaderi^24^ used DPV to apply silver nanodendrimers supported by graphene nanosheets in the linear range of 1–100 μM with an LOD of 0.8 μM in the detection of imidacloprid.

A number of other researchers have developed colorimetric sensors based on AuNPs to detect imidacloprid. Tan et al*.*^1^ modified AuNPs with piperidine-calix [4] arene and detected imidacloprid in the linear range of 0.1–1,000 μM with an LOD of 5 μM. Zhang et al*.*^11^ used an ionic liquid mixture of AuNPs, formed using NaBH_4_ as a reducing agent for the studied analyte in the linear range of 0.01–1,000 μM and an LOD of 0.5 μM.

In the present work, the colorimetric detection of imidacloprid was performed using a GQD/Au (III) sensor without the addition of any toxic reducing agent. Therefore, the current study applied a sustainable colorimetric sensor to the detection of imidacloprid without the complexity that is the main drawback of systems described in previous reports. Also, GQD/Au (III) sensor detects the analyte in the linear range of 0.01–1 ppm (0.039–3.9 μM) with the LOD of 0.007 ppm (0.027 μM). Moreover, the response time of the sensor was quicker than that of the other studies (Table 1).

**Table 1:** Comparison the performance of some methods for detecting imidacloprid.

Methods/materials	Linear range (μM)	LOD (μM)	References
Cyclic voltammetry/*β*-CDP/rGO/GCE	1–150	0.1	^23^
Differential pulse voltammetry/*β*-CDP/rGO/GCE	0.05–150	0.02	^23^
Differential pulse voltammetry/silver nanodendrimers supported by graphene nanosheets	1–100	0.8	^24^
Colorimetry/piperidine-calix [4] arene modified AuNPs	0.1–1,000	5	^1^
Colorimetry/ionic liquid functionalized AuNPs	0.01–1,000	0.5	^11^
Colorimetry/GQDs/Au (III)	0.039–3.9	0.027	This work

## Conclusion

In conclusion, we demonstrated that a GQD/Au^3+^ sensor can recognize the presence of imidacloprid in standard and real cucumber samples by the formation of AuNPs, which change the color of the solution. This phenomenon was the result of Au^3+^ reduction to gold nanoparticles promoted with GQD/imidacloprid. It was also found that increasing λ_max_ with incremental increases in imidacloprid concentration increased the AuNP particle sizes. The developed sensor determined imidacloprid in the linear range of 0.01–1 ppm with an LOD of 0.007 ppm. Moreover, the calibration curve was linear, with an R^2^ of 0.91. On the basis of these color changes in the mixture of GQD/Au^3+^/imidacloprid, a simple and fast colorimetric sensor was designed for the determination of imidacloprid in cucumber fruits. This chemosensor is at the stage of research, but it can have a color template according to different concentrations of pesticide near the sensor for commercial purposes. It can be used as a deployable and on-line device in the field if the sample preparation procedure can be removed. Currently, the sample extraction makes it difficult to use in the farm and limits it to the laboratory cases. In conclusion, the novel sensor has some important advantages, including ease of sensor preparation, sustainability of the sensor, fast response time, and detection in the absence of any auxiliary compound.

## Material and methods

### Materials

The Standard of imidacloprid (C_9_H_10_ClN_5_O_2_, 10 mg, ≥ 95% purity grade) was bought from Sigma-Aldrich Laborchemikalien GmbH, Seelze, Germany. The commercial imidacloprid 35% SC was purchased from Indogulf CropSciences Ltd., India. The pesticides penconazole 20% EW and Chlorpyrifos 40.8% EC were procured from Oxin Chem Co. and Golsam Gorgan Chemicals Co., respectively. Infinito 68.75 SC and Previcur energy 84% SL fungicides were bought from Bayer CropSciences Ltd., Germany. The chemicals acetonitrile HPLC-grade (C_2_H_3_N) and magnesium sulfate anhydrous (MgSO_4_) were purchased from Lichrosolv Ltd., Germany, and Scharlau Chemicals Ltd., Spain, respectively. Sodium chloride (NaCl) was bought from Pars Faraso Co., Iran. Citric acid anhydrous (C_6_H_8_O_7_) was purchased from Scharlau Chemicals Ltd., Spain. Chloroauric acid trihydrate (HAuCl_4_·3H_2_O) was procured from Shanghai Worldyang Chemical Company. All solutions were prepared using deionized water.

### Apparatus

The instruments used in the present research included an ultra-sonication device (Eurosonic 4D, Euronda Co., Italy), a centrifuge (Model Mikro 22R, Hettich Zentrifugen Co., Germany), a high-performance liquid chromatography apparatus (1100 Series, Agilent Ltd., USA), a box furnace for pyrolysis processing, a digital scale (Model CP124S, Sartorius Co., Germany), a magnetic stirrer, a UV–visible spectrophotometer (Biowave II, Biochrom WPA Ltd., UK), a Fourier transform infrared (FT-IR) spectrometer (Nicolet 510, Thermo Fisher Scientific Inc., USA), a nanoparticle analyzer (SZ-100, Horiba Scientific Co., Japan), and a transmission electron microscope (Philips CM100 BioTWIN, Philips/FEI Corporation, Eindhoven, Holland).

### The cultivation of cucumbers in the greenhouse

The cucumber seeds cv. “*Nagene*” were cultivated in July 2018 at Urmia University in a greenhouse at a temperature of 25 ± 5 °C and a humidity of 70 ± 5%. The seeds were planted in a clay-loam soil with N, P, and K macro elements of 0.17%, 9.7 ppm, and 518 ppm, respectively. To control aphids and thrips, imidacloprid 35% SC had been sprayed on the cucumber plants and fruits at a dosage of 0.4 per thousand as recommended on the label for fruits and vegetables. The treatment was replicated four times with and without the pesticide. Samples were taken at 1, 2, 4, 8, 15, 21, and 25 days after spraying. Generally, 32 homogeneous samples were collected from the greenhouse and were frozen until the experiments were conducted.

### HPLC analysis

The detection of imidacloprid in vegetables is usually carried out by HPLC due to its low volatility, high polarity, and thermolability^25^. Here, the authors used a QuEChERS (quick, easy, cheap, effective, rugged, and safe) method to prepare samples according to Neufeld et al*.*’s^26^ study; this method has become more popular in the past decade due to its simplicity, low cost, high throughput, speed, and minimal solvent requirement^27^.

To extract the pesticide from real cucumber samples, each cucumber was taken out of the freezer and shredded with a grinder to obtain a homogenous sample that included both flesh and skin. Each sample (5 ± 0.1 g) was weighed accurately and mixed with acetonitrile (5 ml). Then, NaCl (0.5 ± 0.1 g) and MgSO_4_ (2 ± 0.1 g) were added gradually to the sample under ultra-sonication in a water bath. Next, each sample was centrifuged at 3,000 rpm for about 2 min; afterwards, the upper supernatant was separated from the bottom precipitation and kept in a test tube. Finally, the samples were stored at 4 ± 1 °C until the analysis was conducted.

In the present study, a high-performance liquid chromatography apparatus with a diode array detector (HPLC–DAD) was used. The separation of analyte, the procedure for which is reported in detail by Babazadeh et al*.*^28^, was done on an octadecylsilane (ODS) column. After injecting the standard solution or cucumber extracts (20 µl) into the HPLC apparatus, imidacloprid was separated during isocratic elution with the mobile phase at a flow rate of 1 ml/min. The mobile-phase composition was an acetonitrile/phosphate buffer (70:30 v/v) at pH = 3. Finally, imidacloprid was determined at 227 nm and the quantification of the pesticide residue in samples was calculated by comparison with the peak area of the standard solution’s chromatogram at 2.9 min retention time (Supplementary Fig. S7).

### GQD/Au (III) sensor preparation

GQDs were synthesized using pyrolysis in a box furnace according to the method of Dong et al*.*’s^18^ study. First, precursor or C_6_H_8_O_7_ (10 g) was weighed in a vial. Then, pyrolysis was begun in a beaker at approximately 200 ± 10 °C temperature for about 30 min to change the phase of citric acid from solid to liquid and change the color from colorless to pale yellow and then orange. After the synthesized solution had cooled for a few minutes, deionized water (100 ml) was added and the solution was stirred for some minutes until it displayed the completely yellow color that indicates the formation of GQDs. The GQDs were characterized with common methods including UV–Vis spectrophotometry, FT-IR spectroscopy, and transmission electron microscopy (TEM).

For the preparation of the Au (III) solution (1 mM), HAuCl_4_·3H_2_O (34 mg) was added to a volumetric flask and deionized water was added to a total volume of 100 ml. The color of this solution was pale yellow.

### General procedure for colorimetric detection of imidacloprid in standard and real samples

Typically, GQDs (0.70 ml) and Au^3+^ solution (2 ml) were added to test tubes for preparing GQD/Au (III) sensors. Then, the sample insecticide solution or various concentrations of standard solutions (1 ml) between 0.001 and 1,000 ppm were added to the prepared solutions after a few minutes to obtain the solutions used for analysis. After about 10 min of incubation, the color changes were evident and the absorbance spectra were measured in the range of 200–950 nm using a UV–Vis spectrophotometer.

### The interference study

As cucumber plants are infected by various insects and diseases at harvest time such as powdery mildew, downy mildew, damping-off, and thrips in parallel with aphids, so penconazole, infinito, previcur energy, and chlorpyrifos are selected for interference study, respectively. The experiments were carried out using 0.5 ml of each pesticide (20 ppm) in GQD/Au^3+^/imidacloprid combinations.

## Supplementary information

Supplementary Information.

## Data Availability

The datasets generated during and/or analysed during the current study are available from the corresponding author on reasonable request.
