# Supplementary material for: Colorimetric sensing of imidacloprid in cucumber fruits using a graphene quantum dot/Au (III) chemosensor
Source: Sci Rep. 2020 Aug 31;10:14327. doi: 10.1038/s41598-020-71349-4 (PMC7459307; doi:10.1038/s41598-020-71349-4)
Supplement: Supplementary file 1 — Supplementary Information. [file 41598_2020_71349_MOESM1_ESM.pdf]

Supplementary information

Colorimetric sensing of imidacloprid in cucumber fruits using a graphene quantum dot/Au (III) chemosensor

Saeedeh Babazadeh[1], Parviz Ahmadi Moghaddam[1*], Sajjad Keshipour[2*] & Kaveh Mollazade[3]

[1]Department of Mechanical Engineering of Biosystems, Faculty of Agriculture, Urmia University, 11 km Sero Road, Urmia, Iran.

[2]Department of Nanochemistry, Nanotechnology Research Center, Urmia University, Shahid Beheshti St., Urmia, Iran.

[3]Department of Biosystems Engineering, Faculty of Agriculture, University of Kurdistan, Sanandaj, Iran.

*email: p.ahmadi@urmia.ac.ir; s.keshipour@urmia.ac.ir

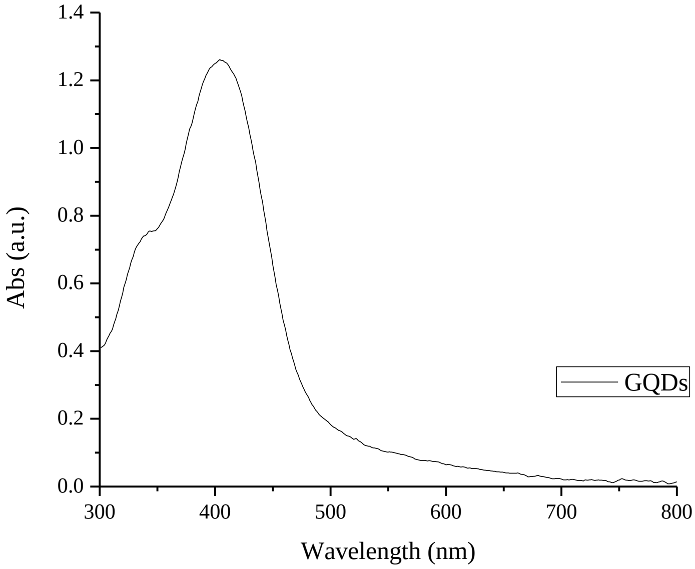

Supplementary Figure S1. The UV-Vis absorption spectrum of GQDs.

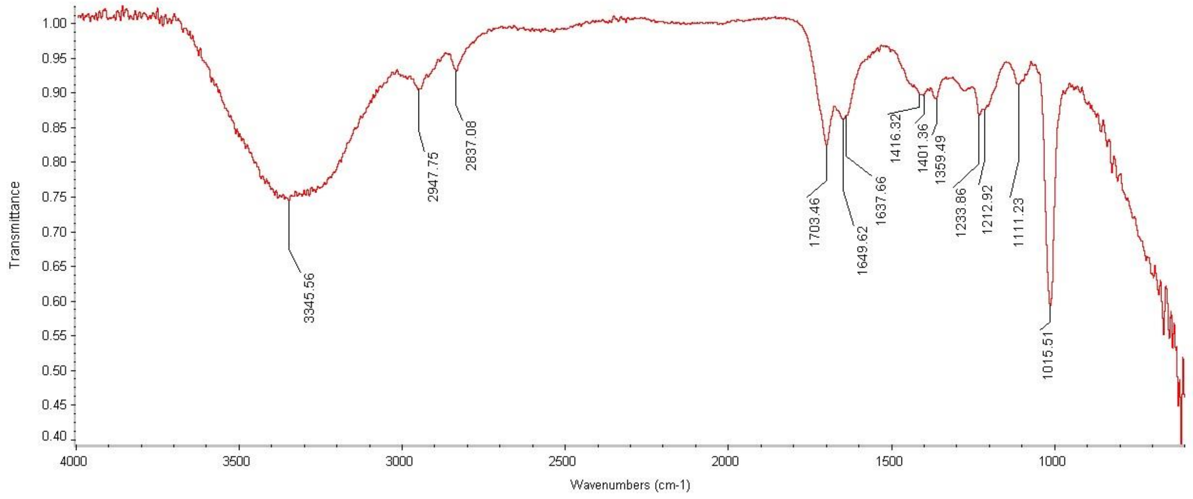

Supplementary Figure S2. FT-IR spectrum of GQDs.

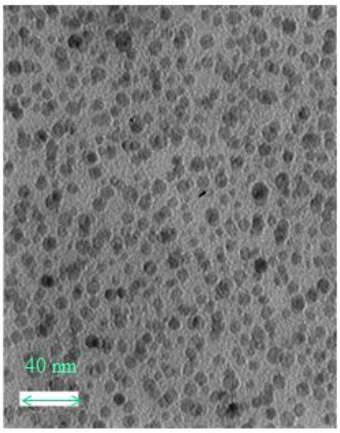

Supplementary Figure S3. TEM image of GQDs (the scale bar is 40 nm).

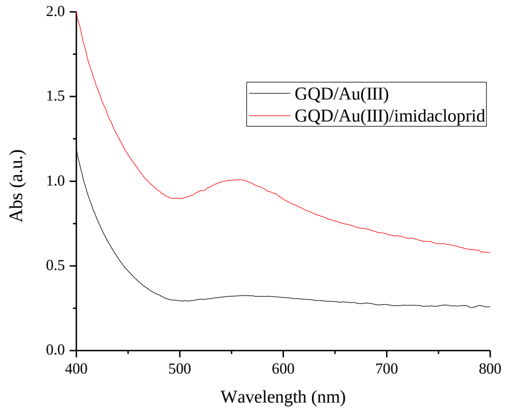

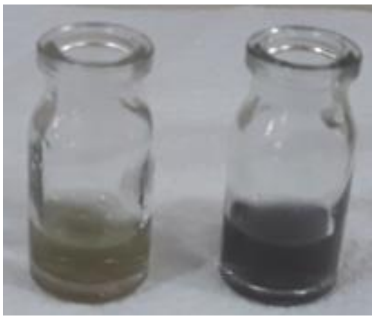

(a) **(b)**

Supplementary Figure S4. UV-Vis spectrum **(a)** and the image **(b)** of GQD/Au (III) in the absence (grey) and presence (reddish grey) of imidacloprid after half an hour.

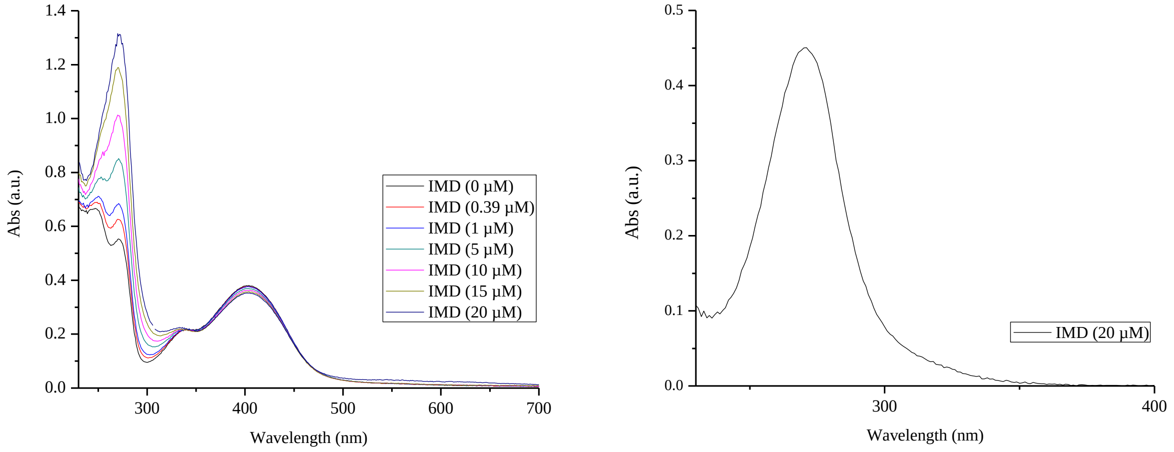

(a) **(b)**

Supplementary Figure S5. (a) The UV-Vis absorbance spectra of GQDs in the presence of imidacloprid (0-20 µM) and **(b)** the absorbance of imidacloprid (IMD) at the concentration of 20 µM.

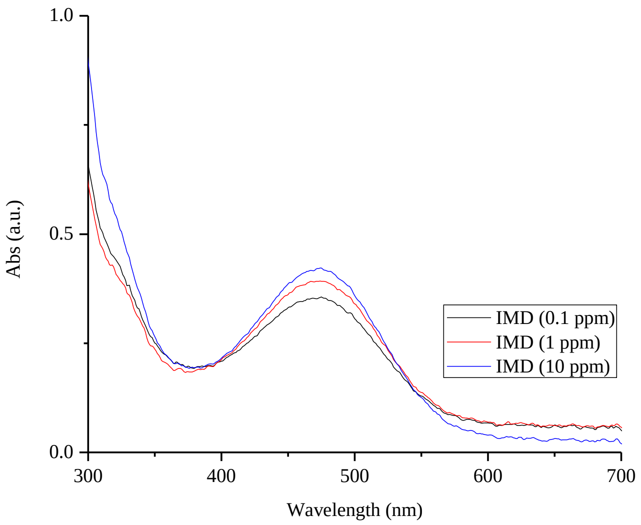

Supplementary Figure S6. The UV-Vis spectra of Au (III) solutions with the addition of three concentrations of imidacloprid (0.1, 1, and 10 ppm).

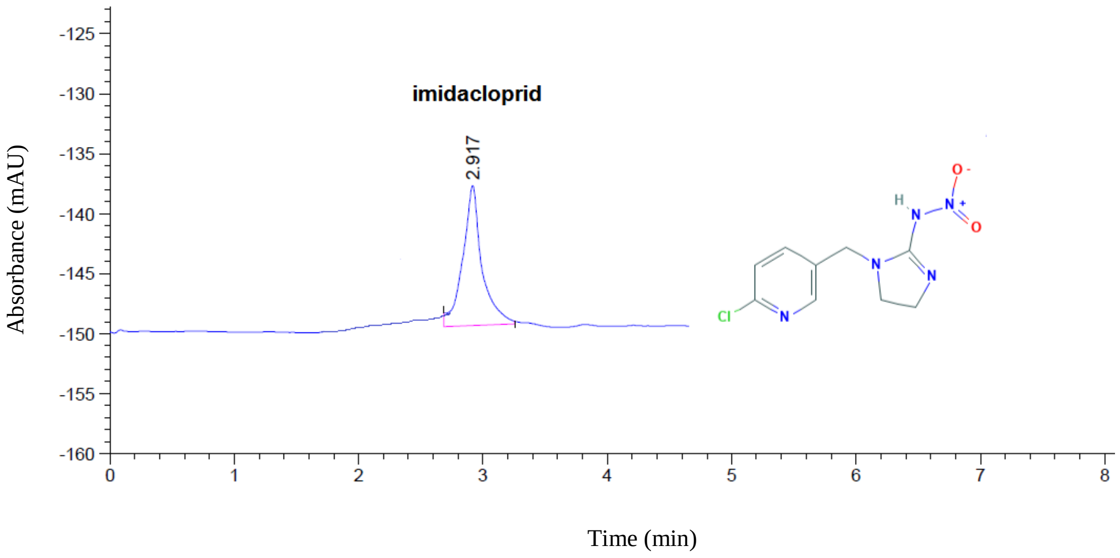

Supplementary Figure S7. The chemical structure and chromatogram of imidacloprid.

Concentration of imidacloprid (ppm)	0.001	0.01	0.1	0.3	0.5	1	2	3	10	100	1000
λ_{max} (nm)	533	534	534	533	532	534	536	538	540	567	-

Supplementary Table S1. The maximum absorbance wavelength (λ_{max}) in various concentrations of imidacloprid.

Sample number	Harvesting time (day)	Imidacloprid (mg/kg)
29	1	1.11
33	2	0.68
37	4	0.30
41	8	0.21
45	15	0.17
49	21	0.14
53	25	0.07

Supplementary Table S2. The mean values of imidacloprid residues detected in cucumber samples by HPLC-DAD.
